# Autism Spectrum Disorder and Particulate Matter Air Pollution before, during, and after Pregnancy: A Nested Case–Control Analysis within the Nurses’ Health Study II Cohort

**DOI:** 10.1289/ehp.1408133

**Published:** 2014-12-18

**Authors:** Raanan Raz, Andrea L. Roberts, Kristen Lyall, Jaime E. Hart, Allan C. Just, Francine Laden, Marc G. Weisskopf

**Affiliations:** 1 Department of Environmental Health; 2 Department of Social and Behavioral Sciences, and; 3 Department of Nutrition, Harvard T.H. Chan School of Public Health, Boston, Massachusetts, USA; 4 Department of Public Health Sciences, University of California, Davis, Davis, California, USA; 5 Channing Division of Network Medicine, Brigham and Women’s Hospital and Harvard Medical School, Boston, Massachusetts, USA; 6 Department of Epidemiology, Harvard T.H. Chan School of Public Health, Boston, Massachusetts, USA

## Abstract

**Background:**

Autism spectrum disorder (ASD) is a developmental disorder with increasing prevalence worldwide, yet has unclear etiology.

**Objective:**

We explored the association between maternal exposure to particulate matter (PM) air pollution and odds of ASD in her child.

**Methods:**

We conducted a nested case–control study of participants in the Nurses’ Health Study II (NHS II), a prospective cohort of 116,430 U.S. female nurses recruited in 1989, followed by biennial mailed questionnaires. Subjects were NHS II participants’ children born 1990–2002 with ASD (*n* = 245), and children without ASD (*n* = 1,522) randomly selected using frequency matching for birth years. Diagnosis of ASD was based on maternal report, which was validated against the Autism Diagnostic Interview-Revised in a subset. Monthly averages of PM with diameters ≤ 2.5 μm (PM_2.5_) and 2.5–10 μm (PM_10–2.5_) were predicted from a spatiotemporal model for the continental United States and linked to residential addresses.

**Results:**

PM_2.5_ exposure during pregnancy was associated with increased odds of ASD, with an adjusted odds ratio (OR) for ASD per interquartile range (IQR) higher PM_2.5_ (4.42 μg/m^3^) of 1.57 (95% CI: 1.22, 2.03) among women with the same address before and after pregnancy (160 cases, 986 controls). Associations with PM_2.5_ exposure 9 months before or after the pregnancy were weaker in independent models and null when all three time periods were included, whereas the association with the 9 months of pregnancy remained (OR = 1.63; 95% CI: 1.08, 2.47). The association between ASD and PM_2.5_ was stronger for exposure during the third trimester (OR = 1.42 per IQR increase in PM_2.5_; 95% CI: 1.09, 1.86) than during the first two trimesters (ORs = 1.06 and 1.00) when mutually adjusted. There was little association between PM_10–2.5_ and ASD.

**Conclusions:**

Higher maternal exposure to PM_2.5_ during pregnancy, particularly the third trimester, was associated with greater odds of a child having ASD.

**Citation:**

Raz R, Roberts AL, Lyall K, Hart JE, Just AC, Laden F, Weisskopf MG. 2015. Autism spectrum disorder and particulate matter air pollution before, during, and after pregnancy: a nested case–control analysis within the Nurses’ Health Study II cohort. Environ Health Perspect 123:264–270; http://dx.doi.org/10.1289/ehp.1408133

## Introduction

Autism spectrum disorder (ASD) is a developmental disorder with increasing reported prevalence worldwide (French et al. 2013). Although genetics plays a strong role in ASD, evidence suggests that environmental exposures, particularly *in utero* or during early life, also affect ASD risk (Grønborg et al. 2013; Hallmayer et al. 2011; Quaak et al. 2013). However, no specific environmental toxicant has been consistently associated with increased risk of ASD.

Air pollution contains various toxicants that have been found to be associated with neurotoxicity and adverse effects on the fetus *in utero* (Crump et al. 1998; Grandjean and Landrigan 2006; Rice and Barone 2000; Rodier 1995; Stillerman et al. 2008). Airborne particles are covered with various contaminants, and have been found to penetrate the subcellular environment and induce oxidative stress and mitochondrial damage *in vitro* (Li et al. 2003; MohanKumar et al. 2008). In rodents, these particles also have been found to stimulate inflammatory cytokine release systemically and in the brain, and alter the neonatal immune system (Hertz-Picciotto et al. 2005, 2008; MohanKumar et al. 2008)—processes that have been implicated in ASD (Depino 2013; Napoli et al. 2013).

Several studies have explored associations of air pollution with ASD, using the U.S. Environmental Protection Agency (EPA) hazardous air pollutant models, distance to freeway, or local models for specific pollutants. These studies suggest increased odds of having a child with ASD with higher exposures to diesel particulate matter (PM) (Roberts et al. 2013; Windham et al. 2006), several metals (Roberts et al. 2013; Windham et al. 2006), criteria pollutants (Becerra et al. 2013; Volk et al. 2013), and some organic materials as well as closer proximity to a freeway (Volk et al. 2011).

Our goal was to explore the association between ASD and exposure to PM during defined time periods before, during, and after pregnancy, within the Nurses’ Health Study II (NHS II), a large, well-defined cohort with detailed residential history. This nested case–control study includes participants from across the continental United States, and exposure was linked to monthly data on two size fractions of PM.

## Methods

*Participants*. The study population included offspring of participants in NHS II, a prospective cohort of 116,430 U.S. female nurses 25–43 years of age when recruited in 1989, followed biennially (Solomon et al. 1997). NHS II participants originally were recruited from 14 states in all regions of the continental United States, but they now reside in all 50 states. The study was approved by the Partners Health Care Institutional Review Board and complied with all applicable U.S. regulations; return of completed questionnaires constituted consent to participate.

In 2005, NHS II participants were asked whether any of their children had been diagnosed with autism, Asperger’s syndrome, or “other autism spectrum,” and 839 women replied affirmatively. In 2007, we initiated a pilot follow-up study, shortly followed by a full-scale follow-up as described previously (Lyall et al. 2012). The follow-up questionnaire included questions about the pregnancy and birth, child’s sex, and diagnosis. NHS II protocol allows re-contacting only the nurses who responded to the most recent biennial questionnaire. Thus, this follow-up was attempted with the 756 mothers of ASD cases for whom this was the case. Mothers who reported having more than one child with ASD were directed to report about the youngest one. Controls were selected from among parous women not reporting a child with ASD in 2005. For each case mother, controls were randomly selected from among those women who gave birth to a child in a matching birth year, to yield a total of 3,000 controls. Six hundred thirty-six (84%) mothers of cases and 2,747 (92%) mothers of controls responded; 164 women (including 51 case mothers) declined to participate.

For the current study, only children whose estimated conception month was June 1989 or later were included because nurses’ addresses before this month were unknown. Of the 265 children reported to have an ASD diagnosis who met this criterion we excluded 4 for whom ASD was not confirmed by the mother on the follow-up questionnaire, and another 2 with genetic syndromes associated with ASD (*n* = 1 Down syndrome; *n* = 1 Rett syndrome). The remaining 259 children were classified as ASD cases. There were 1,640 control children who met the conception month criterion. We further excluded participants missing PM data because their addresses could not be geocoded (8 cases and 30 controls), controls who were reported to have ASD on the 2009 questionnaire (*n* = 9), and children missing data on birth month (6 cases and 79 controls). The final study sample included 245 cases and 1,522 controls born 1990 through 2002. The average (± SD) year of diagnosis of the ASD cases was 1999 ± 3.3. None of these children were reported to have been adopted. Of 188 ASD cases with data on ASD in siblings, 7.4% were reported to have a sibling with ASD. Analyses excluding those 7.4% were similar to analyses including all children and are therefore not reported.

*Case validation*. ASD diagnosis was validated by telephone administration of the Autism Diagnostic Interview–Revised (ADI-R) (Lord et al. 1994) in a subsample of 50 cases randomly selected from mothers who indicated on our follow-up questionnaire willingness to be contacted (81% of all case mothers). In this sample, 43 children (86%) met full ADI-R criteria for autistic disorder [which is stricter than the broader “autism spectrum disorder” of the current DSM-V (*Diagnostic and Statistical Manual of Mental Disorders, 5th Edition*) criteria, or other autism spectrum disorders including PDD-NOS (pervasive developmental disorder not otherwise specified) or Asperger syndrome of DSM-IV criteria], defined by meeting cutoff scores in all three domains (social interaction, communication and language, restricted and repetitive behaviors) and having onset by 3 years of age. The remaining individuals met the onset criterion and communication domain cutoff and missed the autistic disorder cutoff by one point in one domain (*n* = 5; 10%), or met cutoffs in one or two domains only (*n* = 2; 4%), thus indicating presence of ASD traits [for further details on scoring of ADI-R, see Lord et al. (1994)]. In addition, Social Responsiveness Scale (SRS) scores (Constantino et al. 2000), obtained for approximately 90% of eligible cases, also indicated accuracy of case ascertainment. Although it is not a clinical diagnostic instrument, the SRS is a widely used measure of social functioning and autistic traits, and has been shown to have excellent validity as compared to ADI-R and ADOS (Autism Diagnostic Observation Schedule) (Constantino et al. 2003). Among our ASD cases, 93% met the SRS cutoff for ASD. In contrast, 93% of controls completing the same measure fell within the normative range. Therefore, both ADI-R and SRS scores support reliable ASD case ascertainment in our population. For all analyses only the maternal reports were used for determination of ASD status.

*Exposure assessment*. Residential locations of the nurses were determined from the mailing addresses used for the biennial NHS II questionnaire. Monthly ambient exposure predictions of airborne particulate matter with an aerodynamic diameter ≤ 10 μm (PM_10_) and ≤ 2.5 μm (PM_2.5_) were generated from nationwide expansions of previously validated spatiotemporal models (Yanosky et al. 2008, 2009, 2014). The models use monthly average PM_10_ and/or PM_2.5_ data from the U.S. EPA’s Air Quality System (http://www.epa.gov/ttn/airs/airsaqs/), a nationwide network of continuous and filter-based monitors, as well as monitoring data from various other sources. The models also incorporated information on several geospatial predictors including distance to road, population density, point sources (e.g., power-generating utilities, waste combustors), elevation, and meteorology. All data were used in generalized additive statistical models (Yanosky et al. 2008) with smoothing terms of space and time to create separate PM prediction surfaces for each month. Because monitoring data on PM_2.5_ are limited before 1999, PM_2.5_ in the period before 1999 was modeled using data on PM_10_ and visibility data at airports (Yanosky et al. 2009, 2014). PM_10–2.5_ predictions were calculated as the difference between monthly PM_10_ and PM_2.5_ predictions. These models provide estimates for any geolocation in the conterminous United States by monthly intervals. The models also have been shown to have low bias and high precision: The normalized mean bias factor for PM_2.5_ is –1.6%, and the absolute value of the prediction errors is 1.61. For PM_2.5–10_ these values are –3.2% and 4.18, respectively (Yanosky et al. 2014).

For each child, we estimated exposures to PM_2.5_ and PM_10–2.5_ before, during, and after pregnancy by averaging monthly concentrations for the mother’s residential address during the relevant months. The months of pregnancy were determined from the child’s birth month and gestational age at birth, as reported by the mother. Exposures to PM during each pregnancy trimester were calculated similarly.

*Covariates*. The following covariates, all associated with autism in previous studies, were included in multivariable models: child’s birth year, birth month, and sex, maternal age at birth, paternal age at birth, and median census tract income in the birth year. Among these variables, only census tract income (1.5%) and paternal age (10.6%) had missing data. We used the missing indicator method for missing data. We conducted sensitivity analyses to evaluate the influence of adjusting for gestational factors (premature birth, birth weight, gestational diabetes, preeclampsia), smoking during pregnancy, state, marital status, median census house value, paternal education, and maternal grandparents’ education. All covariate data except for census variables were from maternal self-report.

*Statistical analyses*. Logistic regression models were used to estimate odds ratios (OR) and 95% confidence intervals (CI) of ASD by PM exposures modeled both using PM quartiles and as continuous variables, in separate models. Exposures to different PM size fractions were examined in separate models, and also together in a single model.

For nurses who moved residence between two questionnaires straddling pregnancy, we did not know the exact date of moving. Therefore, we conducted separate analyses for exposures assigned assuming the nurse was at the earlier address during the whole intervening period (prepregnancy address) or at the later address during the whole period (postpregnancy address). In addition, to reduce misclassification of exposure, we conducted analyses that were limited to those mothers for whom the pre- and postpregnancy addresses were identical [160 cases (65%) and 986 controls (65%), referred to here as “nonmovers”].

To examine temporal specificity of any associations between PM and ASD, we considered the association with PM_2.5_ exposure during the 9 months before pregnancy, the pregnancy period, and the 9 months after birth. These examinations were restricted to nonmovers with complete data for all exposure periods, and each time period was considered independently, and then also in a single model that included all three time periods simultaneously. Because of differences in ASD rates by sex and prior suggestions that air pollution effects may be specific to boys, we *a priori* decided to also examine associations stratified by sex of the child. For simplicity, we did this only among the children whose mothers did not move during pregnancy. We used SAS version 9.3 (SAS Institute Inc., Cary, NC) for data extraction, and R version 3.0.1 (http://www.r-project.org/foundation/) for Linux-gnu for analyses. All analyses were conducted at the 0.05 alpha level.

## Results

ASD cases were more likely to be male, to have been exposed to maternal preeclampsia or maternal smoking during gestation, and to be missing data on premature birth compared with controls (Table 1). The median (25th–75th percentile) year of birth for cases and controls was the same: 1993 (1991–1996). As expected given time trends in air pollution, control children born in earlier years were more likely to be in higher PM_2.5_ quartiles. Census income and parental age also decreased slightly, but generally steadily by exposure, whereas there was little clear pattern of difference by exposure for other variables (Table 2).

**Table 1:** Study population characteristics by ASD status, Nurses’ Health Study II.

Characteristic	Cases (*n *= 245)	Controls (*n *= 1,522)
Male sex [*n* (%)]	209 (85)	793 (52)
Year of birth [median (IQR)]	1993 (5)	1993 (5)
Maternal age at birth (years) (mean ± SD)	34.0 ± 4.0	33.7 ± 3.7
Paternal age at birth (years) (mean ± SD)	36.8 ± 5.3	36.3 ± 4.9
Median census income ($1,000) [median (IQR)]	63 (26)	61 (27)
Median census house value ($1,000) [median (IQR)]	144 (108)	136 (98)
Birth weight (lbs) (mean ± SD)	7.1 ± 1.5	7.2 ± 1.3
Husband’s/partner’s education [*n* (%)]
High school	33 (13)	208 (14)
2-year college	45 (18)	218 (14)
4-year college	79 (32)	537 (35)
Graduate school	74 (30)	501 (33)
Missing	14 (6)	58 (4)
Marital status [*n* (%)]
Married	186 (76)	1,159 (76)
Never married	51 (21)	269 (18)
Other	8 (3)	94 (6)
Premature birth [*n* (%)]
Yes	44 (18)	227 (15)
No	142 (58)	1,137 (75)
Missing	59 (24)	158 (10)
Gestational diabetes [*n* (%)]
Yes	17 (7)	87 (6)
No	189 (77)	1,222 (80)
Missing	39 (16)	213 (14)
Preeclampsia [*n* (%)]
Yes	13 (5)	43 (3)
No	193 (79)	1,266 (83)
Missing	39 (16)	213 (14)
Smoking during pregnancy [*n* (%)]
Yes	22 (9)	50 (3)
No	160 (65)	1,099 (72)
Missing	63 (26)	373 (25)
IQR, Interquartile range.		

**Table 2:** Control population characteristics by pregnancy PM_2.5_ quartile, Nurses’ Health Study II (*n* = 1,522 controls).

Characteristic	Quartile [μg/m^3^ (range)]			
	1st (5.24–12.3)	2nd (12.4–14.5)	3rd (14.6–16.7)	4th (16.7–30.8)
*n*	397	376	375	374
Male sex (*n* (%)]	208 (52)	203 (54)	192 (51)	190 (51)
Year of birth [median (IQR)]	1995 (5)	1994 (4)	1993 (4)	1992 (3)
Maternal age at birth (years) (mean ± SD)	34.3 ± 3.8	34.0 ± 3.8	33.5 ± 3.6	32.7 ± 3.6
Paternal age at birth (years) (mean ± SD)	37.4 ± 5.1	36.5 ± 4.9	36.1 ± 5.0	35.2 ± 4.5
Median census income ($1,000) [median (IQR)]	62 (31)	64 (28)	61 (26)	58 (24)
Median census house value ($1,000) [median (IQR)]	137 (107)	144 (104)	135 (96)	128 (82)
Birth weight (lbs) (mean ± SD)	7.2 ± 1.3	7.2 ± 1.2	7.1 ± 1.3	7.2 ± 1.3
Premature birth (*n* (%)]
Yes	56 (14)	57 (15)	52 (14)	62 (17)
No	298 (75)	282 (75)	275 (73)	282 (75)
Missing	43 (11)	36 (10)	48 (13)	30 (8)
Gestational diabetes (*n* (%)]
Yes	18 (5)	27 (7)	21 (6)	21 (6)
No	314 (79)	303 (81)	299 (80)	306 (82)
Missing	65 (16)	46 (12)	55 (15)	47 (13)
Preeclampsia (*n* (%)]
Yes	12 (3)	9 (2)	8 (2)	14 (4)
No	320 (81)	321 (85)	312 (83)	313 (84)
Missing	65 (16)	46 (12)	55 (15)	47 (13)
Smoking during pregnancy (*n* (%)]
Yes	17 (4)	17 (4)	21 (5)	17 (4)
No	323 (73)	313 (71)	308 (70)	315 (71)
Missing	102 (23)	112 (25)	112 (25)	110 (25)
IQR, interquartile range.				

**Figure 1:**
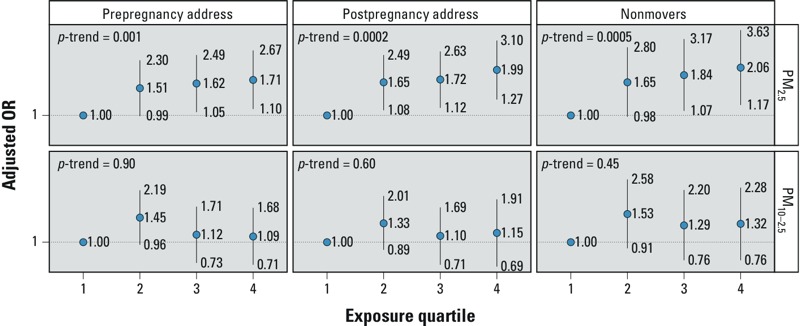
ORs (95% CIs) for ASD by quartile of PM exposure. ORs are adjusted for child sex, year of birth, month of birth, maternal age at birth, paternal age at birth, and census income. There were 245 cases and 1,522 controls in analyses using pre- and postpregnancy addresses. Prepregnancy address is the last known residential address before conception. Postpregnancy address is the first known residential address after birth. Nonmovers are those participants for whom prepregnancy and postpregnancy addresses were the same [cases = 160 (65%), controls = 986 (65%)]. *p-*Trend, *p*-values from models of exposures as continuous variables. The number of cases (including movers) by quartiles from low to high: 45, 66, 66, 68; controls: 397, 376, 375, 374. PM_2.5_ quartile ranges (μg/m^3^): 5.24–12.3, 12.4–14.5, 14.6–16.7, 16.7–30.8; PM_10–2.5_ quartile ranges (μg/m^3^): 1.9–6.7, 6.8–8.9, 9–11.9, 12–49.4.

The average (± SD) levels of PM_2.5_ and PM_10–2.5_ during pregnancy were 14.6 ± 3.3 and 9.9 ± 4.9 μg/m^3^, respectively. Although PM_10–2.5_ did not show a clear and consistent association with ASD, PM_2.5_ was associated with ASD regardless of the address used for the PM estimation (Figure 1). Among nonmovers, for whom misclassification of exposure because of an address change is reduced, the OR was 2.06 (95% CI: 1.17, 3.63) in the 4th quartile, compared with the 1st quartile. The results were also similar when analysis was limited to nonmovers and used continuous PM estimates, with an OR of 1.57 (95% CI: 1.22, 2.03) per interquartile range (IQR) increase in PM_2.5_ (4.42 μg/m^3^), and little association with PM_10–2.5_ [OR = 1.07 per PM_10–2.5_ IQR (5.15 μg/m^3^); 95% CI: 0.89, 1.28]. When PM_2.5_ and PM_10–2.5_ were in the same model together, the difference between the two was greater: OR = 1.61 per PM_2.5_ IQR (95% CI: 1.22, 2.12); OR = 0.96 per PM_10–2.5_ IQR (95% CI: 0.79, 1.17). The association with PM_2.5_ among nonmovers was slightly stronger for boys (OR = 1.73; 95% CI: 1.29, 2.31) than for girls (OR = 1.12; 95% CI: 0.59, 2.12), but there were only 23 nonmover girls with ASD (137 nonmover boys), and the interaction *p*-value was 0.17.

When estimating the association with PM_2.5_ exposure during the 9 months before pregnancy, the pregnancy period, and the 9 months after birth, all restricted to nonmovers with exposure estimates for all three exposure periods, the associations with exposures before or after the pregnancy were lower compared with the association with exposure during pregnancy (Table 3). The partial correlation of PM_2.5_ during pregnancy with PM_2.5_ during the 9 months before or after pregnancy was 0.85 and 0.83, respectively. When we included all three PM_2.5_ exposure periods together in a mutually adjusted model, ASD was significantly associated only with exposure during the pregnancy period (Table 3). This pattern did not change after further restriction to women who did not move during the whole period from 9 months before conception to 9 months after birth (data not shown).

**Table 3:** ORs (95% CI) for ASD per IQR increase in PM_2.5_ levels in different time periods, nonmovers only.*^a^*

Exposure period	OR (95% CI) per 4.40 μg/m^3^ PM_2.5_		
	Unadjusted	Adjusted^*b*^	Mutually adjusted^*c*^
9 months before conception	1.20 (0.98, 1.47)	1.32 (1.04, 1.69)	0.83 (0.58, 1.19)
Whole pregnancy	1.37 (1.09, 1.71)	1.50 (1.16, 1.94)	1.63 (1.08, 2.47)
9 months after birth	1.19 (0.96, 1.49)	1.29 (1.00, 1.67)	0.96 (0.65, 1.40)
^***a***^Restricted to nonmovers who also have data on all exposure periods (158 cases, 977 controls). ^***b***^Adjusted for child sex, year of birth, month of birth, maternal age at birth, paternal age at birth, census income. ^***c***^Mutually adjusted for other two exposure periods, as well as all other covariates listed above.			

**Figure 2:**
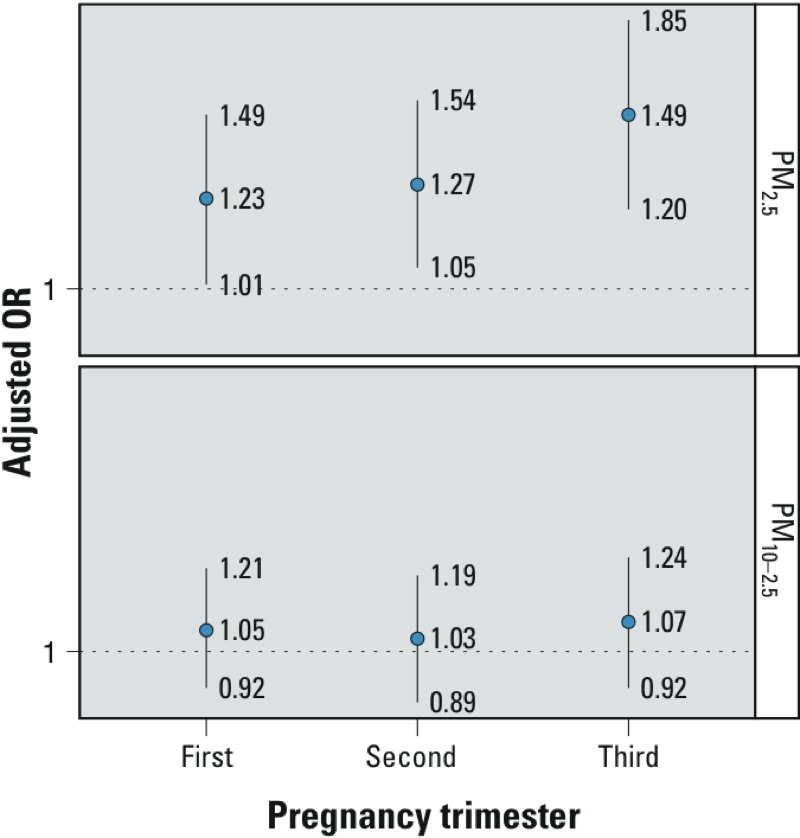
ORs for ASD with exposure to particulate matter during pregnancy trimesters. ORs are adjusted for child sex, year of birth, month of birth, maternal age at birth, paternal age at birth, and census income. The analyses are limited to nonmovers only (i.e., those for whom prepregnancy and postpregnancy addresses were the same). Cases, *n *= 160, controls *n *= 986.

When examining trimester-specific associations in nonmovers, exposure to PM_2.5_ was associated with ASD in all three trimesters, but PM_10–2.5_ was not associated with ASD in any of the trimesters (Figure 2). The highest association with PM_2.5_ was seen in the third trimester (OR = 1.49 per PM_2.5_ IQR; 95% CI: 1.20, 1.85) (Figure 2). In a model with all trimesters mutually adjusted, the only statistically significant association was seen with PM_2.5_ in the third trimester (OR = 1.42; 95% CI: 1.09, 1.86), whereas exposure during the first and second did not show associations (OR = 1.06; 95% CI: 0.83, 1.35, and OR = 1.00; 95% CI: 0.78, 1.30, respectively). When third-trimester PM_2.5_ and PM_10–2.5_ were in the same model together, the difference between the two was greater: OR = 1.50 per PM_2.5_ IQR (95% CI: 1.19, 1.89); OR = 0.89 per PM_10–2.5_ IQR (95% CI: 0.81, 1.19).

ORs and CIs were comparable in separate analyses excluding premature births, or participants missing data on census tract income, or paternal age (data not shown). Adjusting for PM_10–2.5_ also resulted in comparable estimates for PM_2.5_ (data not shown). Results were also similar in models adjusted for (each in a separate model): gestational variables (premature birth, birth weight, gestational diabetes, preeclampsia), smoking during pregnancy, census tract house value, state, marital status of the nurse, or husband’s/partner’s education or maternal grandparents’ education (data not shown). In addition, models limited to either mothers with white race/ethnicity (95% of the nurses) or children who had a full-term pregnancy (i.e., excluding premature births and those with missing data on this variable) showed comparable estimates (data not shown).

## Discussion

In our nested case–control study of nurses from across the continental United States, ambient PM_2.5_ concentrations during pregnancy were significantly associated with having a child diagnosed with ASD. Importantly, the association we found appeared specific to PM_2.5_ during pregnancy; PM_2.5_ exposure before or after pregnancy showed weaker associations with ASD, and PM_10–2.5_ during pregnancy showed little association with ASD. In a model mutually adjusted for all three exposure periods, only the pregnancy period was associated with ASD. The change in the ORs with mutual adjustment did not appear to be an artifact of collinearity because the precision of the mutually adjusted model was not substantially lower than the single exposure model (e.g., CI widths for an IQR change in PM_2.5_ during pregnancy of 2.3 vs. 1.7, respectively). The 95% CIs were not notably larger in this analysis, suggesting that collinearity was not a significant problem. Moreover, during pregnancy we found the association to be specifically with the third-trimester exposure in models that included exposure in all trimesters together. The specificity of the association to the prenatal period is in line with several other lines of evidence that suggest a prenatal origin of ASD, including data on differences in brain cytoarchitecture in brains of children with ASD (McFadden and Minshew 2013; Stoner et al. 2014) and associations between maternal exposure to teratogens during pregnancy and ASD (Rodier 1995). Our results also suggest an association predominantly in boys, but this finding should be interpreted with caution, given the small number of girls with ASD in our sample.

These results generally agree with previous studies. A report from the CHildhood Autism Risks from Genetics and the Environment (CHARGE) study among 304 ASD cases and 259 controls, in several areas in California, used residential address history reported by parents to calculate distance to roads as a proxy for traffic-related air pollution exposure and found increased risk for ASD among women who lived in proximity to a freeway (Volk et al. 2011). Further analysis of the CHARGE study group in a subset of 279 cases and 245 controls using data from the U.S. EPA Air Quality System suggested positive associations of ASD with traffic-related air pollution during pregnancy, and specifically with PM_2.5_ (Volk et al. 2013). ASD was also associated with pregnancy exposure to PM_10_, and—in contrast to our results—the association with traffic-related air pollution exposure during the first year of life was higher than that found for the exposure during pregnancy. In the CHARGE study, associations were also seen with exposures in the year after birth that were about as strong as exposures during pregnancy. Our findings suggested a weaker association with postpregnancy exposure that was essentially null in models that included exposure during all time periods. In the CHARGE study, however, the pregnancy and postpregnancy exposure periods were not included together in the same regression model.

Another study, from Los Angeles (LA) County, used birth certificate address and ASD cases identified from the Department of Developmental Services in California (Becerra et al. 2013). Using exposure data from the nearest monitoring stations and from a land use regression model (Su et al. 2009), they found a positive association between PM_2.5_ exposure and autism (OR per 4.68 μg/m^3^ PM_2.5_ = 1.15; 95% CI: 1.06, 1.24 in a model of exposure over the entire pregnancy and also adjusted for ozone levels). There was not a consistent association with PM_10_. The LA study included many more ASD cases than any of the other studies, so the effect estimate could represent a more stable estimate of the true effects of PM. Alternatively, differences in the composition of PM in the LA area could result in smaller effects. Other differences in study design could also have led to smaller effect sizes in the LA study. The case definition was a primary diagnosis of autistic disorder, the most severe among ASD diagnoses, and the association with PM could be preferentially with milder forms of ASD. Slightly more measurement error from using a nearest monitor exposure assignment approach or addresses from the birth certificate could have biased results toward the null. Smaller associations in that study could also have occurred if there was under-ascertainment of cases among children of more highly exposed mothers. Lower socioeconomic status has been associated with under-ascertainment in ASD registries such as that used in the LA study (Kalkbrenner et al. 2012). Although estimates were not much different when the sample was stratified by education level, if residual socioeconomic differences were associated with PM_2.5_ exposures (lower socioeconomic status with higher PM_2.5_) this could lead to bias toward the null because the controls included all birth certificates in the region. The importance of the environment in the development of ASD was recently implicated in a comparison of concordance rates between monozygotic and dizygotic twins that found that the shared environment accounted for 58% (95% CI: 30, 80%) of the broader autism phenotype (Hallmayer et al. 2011). In line with these findings, a comparison of sibling ASD recurrence risk in a different population revealed a much higher rate of ASD recurrence in half-siblings with the same mother (2.4; 95% CI: 1.4, 4.1) compared with half-siblings with the same father (1.5; 95% CI: 0.7, 3.4) (Grønborg et al. 2013). This finding may be attributed either to maternal factors affecting the *in utero* environment or to common mitochondrial DNA.

Exposure to high levels of environmental toxicants during pregnancy might interfere with normal *in utero* processes of brain development, such as neurogenesis, cell proliferation, cell differentiation, and apoptosis (Rice and Barone 2000; Rodier 1995). PM_2.5_ and especially ultrafine particles (< 0.1 μm in diameter) were shown to penetrate the subcellular environment and to induce strong oxidative stress and mitochondrial damage *in vitro* (Li et al. 2003). These effects were associated with the organic carbon and polycyclic aromatic hydrocarbon contents of the particles (Li et al. 2003). *In vivo* studies in rodents have also shown that PM_2.5_ activates the stress axis, involves microglial activation, and causes production of pro-inflammatory cytokines in the brain (MohanKumar et al. 2008). In one study, increased mitochondrial DNA damage, possibly caused by reactive oxygen species, was found to be more common in 67 children with ASD than in 46 typically developing children (Napoli et al. 2013).

PM_2.5_ may alter the development of the neonatal immune system. In a study of 1,397 children in the Czech Republic, gestational exposures to PM_2.5_ and polycyclic aromatic hydrocarbons were associated with reduction in T cells and an increase in B lymphocytes in neonatal cord blood (Hertz-Picciotto et al. 2005). Early activation of the immune system and neuroinflammation have been found to be associated with ASD in humans (Atladóttir et al. 2010; Careaga et al. 2013; Depino 2013; Gadad et al. 2013; Libbey et al. 2005; Patterson 2011) and in animal models of autism (Gadad et al. 2013; Libbey et al. 2005; Patterson 2011), and this has been proposed as a possible mechanism by which environmental toxicants could increase the risk of ASD (Hertz-Picciotto et al. 2008). Furthermore, a recent transcriptomic comparison of postmortem brain tissues of individuals with ASD (*n* = 19) and controls (*n* = 17) taken from the Autism Tissue Project, the Harvard Brain Bank, and the MRC London Brain Bank for Neurodegenerative Disease, revealed involvement of genes related to synaptic and neuronal signaling dysfunction, and also microglial and immune dysregulation (Voineagu et al. 2011). The implicated genes related to synaptic and neuronal signaling dysfunction, compared with those related to immune changes, had more overlap with genes identified in genome-wide association studies (Voineagu et al. 2011). This suggests that expression of immune-related genes in ASD may be driven more by environmental influences than underlying genetic differences.

These processes that could affect neurodevelopment are general in nature, so the question still would remain why there is an association specifically with ASD. However, the ASD phenotype is quite heterogeneous, and ASD can share features with other neurodevelopmental disorders (e.g., intellectual disability). There is some suggestion that exposures to components of air pollution can also affect neurodevelopment more generally (Perera et al. 2009; Suglia et al. 2008). Determining the range of phenotypic profiles associated with maternal PM exposure during pregnancy would be of interest in future studies. Another interesting direction for future research would be to determine whether the association between PM and ASD is different among children who have one or more siblings with ASD.

A limitation of our study is that we did not have the exact dates on which mothers changed addresses. Thirty-five percent of the nurses (both cases and controls) changed their residential address between the last questionnaire before pregnancy and the first questionnaire after delivery. However, we found statistically significant associations with pregnancy PM when the exposure for movers was based either on pre- or postpregnancy address. When we reduced exposure misclassification by analyzing the smaller sample of nonmovers, the association between ASD and PM_2.5_ was stronger. We also did not have information on how much time the nurses actually spent at their residential addresses, nor did we have information about their work addresses. Error from this source, however, would not affect the estimates of PM at the residential address and so would not create an association with residential PM levels where an association with PM exposure did not exist. Information was also unavailable on indoor air pollution exposures and sources. In addition, ASD diagnoses in the study were based on parental reporting. As medically trained professionals, however, nurse mothers’ reporting of ASD is likely to be reliable, a supposition supported by our validation study using the ADI-R.

Strengths of our study include the wide geographic distribution of the nurses and the nesting within a well-defined cohort, which reduces the likelihood of selection bias. In addition, the specificity of our findings for the pregnancy period places important limitations on possible residual confounding. Specifically, any factor that is not differentially related to PM during pregnancy versus before or after pregnancy is very unlikely to confound our results. Thus, for example, although population density, a choice to take folate supplements during pregnancy, or a host of other potential confounders (Gray et al. 2013; Kalkbrenner et al. 2012) may be related to PM_2.5_ exposure, they would be expected to be equally related to PM_2.5_ exposure before or after pregnancy as during it. But no association with them were seen in mutually adjusted models. In this way, PM_2.5_ exposure before and after pregnancy (because no association is seen with them in mutually adjusted models) acts as a negative control (Flanders et al. 2011; Lipsitch et al. 2010) and rules out confounding by many—even unmeasured—potential confounders. We cannot, however, rule out another pollutant that co-varies with PM_2.5_. Nor can we determine whether there is a particular component of PM_2.5_ that is responsible for the associations we found. PM_2.5_, however, is a complex mixture that may be correlated with other air pollution constituents. In the present study we did not have high temporal and spatial resolution data on other air pollution constituents or on specific PM_2.5_ components to determine whether a specific component is associated with autism.

## Conclusions

Our findings support the possibility of an effect of maternal exposure to air pollution during pregnancy, and especially during the third trimester, on the development of ASD in her child. The results suggest that air pollution is a modifiable risk factor for autism, and reduced exposure during pregnancy could lead to lower incidence of ASD and reduce the substantial, increasing economic burden of ASD on families and on society (Croen et al. 2006; Leslie and Martin 2007; Mandell et al. 2006; Raz et al. 2013; Shimabukuro et al. 2008). Understanding the biological mechanism that may underlie the association by which PM exposure and ASD could provide important insight to ASD pathogenesis.
